# Preventing sexual violence in sport: Determinants of positive coach-bystander behavior

**DOI:** 10.3389/fpsyg.2022.862220

**Published:** 2022-07-20

**Authors:** Helena Verhelle, Tine Vertommen, Gjalt-Jorn Ygram Peters

**Affiliations:** ^1^Forensic Psychology, Thomas More University of Applied Sciences, Antwerp, Belgium; ^2^Social Epidemiology and Health Policy (SEHPO), University of Antwerp, Antwerp, Belgium; ^3^Department of Methods and Statistics, Faculty of Psychology, Open University of the Netherlands, Heerlen, Netherlands

**Keywords:** bystander behavior, sexual violence, sport, safeguarding, intervention

## Abstract

Given their central role and position, coaches are instrumental in creating safe sport environments, especially in preventing sexual violence, but little is known about bystander behaviors, hampering the development of effective bystander programs in the context of sport. To identify determining characteristics of bystander behavior, 1,442 Belgian youth sport coaches completed an online questionnaire on bystander-related attitudes, norms, autonomy beliefs, and self-efficacy using two hypothetical scenarios of sexual violence in the sports club. Data were analyzed using confidence interval-based estimation of relevance (CIBER). A total of 127 coaches had witnessed sexual violence over the past year, most but not all intervened. Experiential attitude expectation, instrumental attitude evaluation, perceived referent behavior and approval, and subskill presence were positively associated with coaches’ intention to intervene. Of the determinants of positive coach-bystander behavior, attitude and perceived norms proved key constituents for programs addressing sexual violence in youth sport. We conclude that interventions aiming at increasing positive affective consequences, reinforcing the sense of group membership, and strengthening the social norm of intervening in case of signs of sexual violence may be most influential to stimulate positive coach-bystander behavior.

## Introduction

Sexual violence is no longer an unspoken issue in the world of youth sport. It is not only a potentially criminal issue, but prevalence estimates also clearly illustrate the problem as a health issue. Mountjoy et al. (2016), p. 3 define sexual violence as “any conduct of a sexual nature, whether non-contact, contact or penetrative, where consent is coerced/manipulated or is not or cannot be given.” In this study, we likewise use the term as an umbrella concept encompassing verbal and non-verbal contact forms of sexual harassment and abuse. Depending on the study designs and measures used, prevalence rates for sexual harassment range from 19% to 92% and for sexual abuse from 2% to 49% (Mountjoy et al., 2016; Bjørnseth and Szabo, 2018). In a recent European study, surveying over 10,000 young adults in six European countries, 32% of the respondents in Flanders (Belgium) reported having experienced at least one form of non-contact sexual violence and 20% reported having experienced at least one form of contact sexual violence within the context of organized sports before the age of 18 (Hartill et al., 2021; Vertommen et al., 2021). In most cases, it did not concern single events but repeated incidents that took place over longer periods of time (Vertommen et al., 2019).

The often severe physical, psychological, social, and societal consequences of such incidents render sexual violence in sport a serious public health issue (Koontz et al., 2021). Yet, despite its high prevalence and impact, signs or incidents of sexual violence in sport are often overlooked or not acted upon. Not intervening or offering help allows the violence to continue, facilitating the perpetrator and thus aggravating and prolonging the victim’s suffering (Cense and Brackenridge, 2001; Spaaij and Schaillée, 2019). The role of bystanders (i.e., individuals that are not directly involved as a victim or perpetrator that have the potential to intervene and amend the situation; Banyard, 2008) can be important in the context of sexual violence.

Banyard et al. (2020) distinguish two types of bystander interventions: reactive and proactive. Reactive bystander actions concern both the negative or positive actions that a person undertakes in response to a high-risk situation (McMahon and Banyard, 2012; Rothman et al., 2019), where negative reactive bystander action is defined as either a lack of action or unhelpful actions (e.g., making fun of the victim, applauding the perpetrator’s conduct) immediately before, during or after an incident of sexual violence. Conversely, positive reactive bystander behavior encompasses all potentially helpful actions directly before, during or after an incident, i.e., at the time of an immediate risk or an ongoing, witnessed event of sexual violence.

Proactive bystander behavior is, in contrast, not restricted to one specific incident but comprises all supporting behaviors aimed at ameliorating or preventing sexual violence in general, (which may, for instance, include taking a course on sexual violence or remaining aware and perceptive of its risk or occurrence; Banyard et al., 2020). In the literature, people who take responsive and helpful actions in emergency situations are also labeled as “actionists” (Rothman et al., 2019), upstanders’ (Ferrans et al., 2012), “defenders” (Pozzoli et al., 2012), or “prosocial bystanders” (Banyard, 2011). In the present study, the terms positive bystander(s) and positive bystander behavior include both reactive and proactive onlookers and interventions.

Positive bystanders play an important role in maintaining a safe environment, and over the last decade the development, implementation, and evaluation of bystander interventions has received more attention in research, policy-making and practice than ever before. By educating people about sexual violence and bystander options, peer norms, attitudes, and beliefs can be changed. Adequately intervening in situations of sexual violence is not only beneficial to the victim and might stop the perpetrator, it also models the desired behavior, potentially encouraging fellow-bystanders to do the same on future occasions. In addition, positive bystander behavior may also reduce the risk of victimization and perpetration in bystanders themselves (Banyard, 2015).

A variety of bystander intervention programs in the context of violence is available (e.g., “Bringing in the bystander,” “Coaching boys into men,” “Green dot”), but only a few focuses on the context in sport. Most were developed in the United States and mainly focus on the prevention of sexual and dating violence on college campuses (e.g., Miller et al., 2012; Palm Reed et al., 2015; Fenton and Mott, 2018). Some of these bystander programs have been systematically reviewed and were found to be effective with small to moderate effects on different components. Some bystander interventions were effective in increasing or promoting actual bystander behavior (Katz and Moore, 2013; Kettrey et al., 2019; Kettrey and Marx, 2019), while others were effective in increasing knowledge, pro-social attitudes and beliefs about sexual violence (Jouriles et al., 2018). The authors concluded that the main reason for those small effects was the use of small samples (Jouriles et al., 2018; Kettrey et al., 2019).

Banyard et al. (2004, 2007), for example, showed that one- or three-session programs had improved attitudes, increased bystander efficacy and knowledge, and, most importantly, engendered prosocial bystander behavior in 389 male and female undergraduates. The recent meta-analysis of Jouriles et al. (2018) confirmed these findings and found a greater effect on bystander attitudes and beliefs for programs with a longer duration, but the effects diminished over time.

Studies regarding perceived norms, which includes the perception of other’s willingness to intervene show that these norms play a significant role in bystander behavior (e.g., Brown and Messman-Moore, 2010; McMahon, 2015; Santacrose et al., 2020). People may be concerned about a violent situation but feel not confident to act, as the group norm implies it is not a problem (Fenton and Mott, 2018).

In their study, Levine et al. (2019) explored key factors involved in violence reduction and bystander interventions. Firstly, they emphasize that intervening in violent or hazardous situations is already the social norm among bystanders, even though interventions may not always be successful. Secondly, the authors stress that people do intervene even when others are present, thus showing that the popular belief that people do not act in the presence of other witnesses is a fallacy, making this a vital message to convey in bystander campaigns. Another major issue they describe is that people often think it is not safe to intervene. However, the researchers found the opposite: the chance of being victimized while intervening is quite low. The final finding they report on concerns the social identity relationship between bystander and victim or perpetrator: bystanders are more likely to intervene in situations involving members of their own social or ethnic group are being victimized (e.g., friends, family, and acquaintances).

To make prevention programs even more effective, Moynihan and Banyard (2008) recommend instructing course participants to not focus on possible perpetrators or victims only but rather on all stakeholders in the organization or environment given the notion that all members of a community have a role in preventing violence or harm and that everyone can become a bystander. Banyard (2015) states that prevention programs should be directed at promoting proactive and reactive behaviors among *all* members of a given community.

When aiming to promote positive coach-bystander behavior, we first need to analyze its constituent parts. Before taking action, any bystander will, consciously or unconsciously, go through several, interlaced stages. First of all, they will have to note and interpret a situation as a problem. Next, they should feel the responsibility to do something about it, for if they do not, they will not take action. When they do experience a sense of responsibility, they will subsequently need to figure out what to do and, if a decision is reached, eventually choose to take action (Banyard, 2011). Each of these constituent stages can be influenced by different environmental conditions and psychological constructs (i.e., behavioral determinants; Bartholomew et al., 2006). A first step in developing successful coach-bystander intervention programs is translating these steps in the context of sport and subsequently gaining insight into the determinants of each sub-behavior.

Knowing that signs of sexual violence in sport are often overlooked or not acted upon (Vertommen et al., 2019), understanding this behavior is crucial. The literature does not provide these insights in this context, therefor this paper aims to identify the determinants that contribute to coach-bystander behavior regarding sexual violence following the Intervention Mapping Protocol. This framework outlines steps, tasks and processes to help develop practical health promotion and education programs through stimulating desirable behaviors (Bartholomew et al., 2006). These insights will inform the development of a dedicated coach-bystander training program to prevent sexual violence in youth sports.

To this end, we adopted the reasoned action approach (RAA; Fishbein and Ajzen, 2010) as a theoretical framework. The RAA has been used multiple times to predict a variety of behaviors, but not in the context of coach-bystander behavior regarding sexual violence. Identifying the essentials predictors of coaches’ intention, constitutes the basis for developing a coach-bystander intervention, and the RAA offers these predictive insights as the theoretical framework links people’s intention to underlying attitudes, norms and beliefs. The practical contribution of this study lays in the next step, where the found determinants will be translated into applications that are part of the actual intervention.

The RAA model builds on the theory of planned behavior (Ajzen, 1991) and the theory of reasoned action (Fishbein and Ajzen, 1975) which explains why people decide to perform (or not to perform) a certain behavior (Fishbein and Ajzen, 2010). The RAA states that the intention to carry out a behavior, such as intervening in case of a violent event, is determined by a person’s attitude toward the behavior, their idea of how relevant others perceive the behavior (perceived norms), and their sense of behavioral control. For people with high intention to intervene, actual and successful execution of the behavior also depends on their abilities and skills, as well as environmental factors. The RAA explicitly does not address automatic behaviors (e.g., habits). Since positive bystander behavior is only infrequently called for, it firmly belongs to the domain of reasoned action.

When behavior is explained by a theory, the variables are often called determinants. A determinant is a psychological variable on global scale, which can be further specified in subdeterminants. Subdeterminants are situated on a lower level of the psychological generality to predict the overarching determinant (Peters and Crutzen, 2018).

Attitude, the first determinant of the RAA, is defined as a person’s subjective evaluation of the consequences of a behavior, conceptualized as favorable or unfavorable. Fishbein and Ajzen (2010) distinguish instrumental and experiential attitude, where in the first the individual sees the outcome as instrumental to their goal (e.g., “If I intervene, this is likely to end the sexual violence.”), whereas experiential attitude is about the anticipated affective consequences of the behavior (e.g., “If I intervene, it is likely that I will feel confident”).

Perceived norms refer to a person’s perception of the approval or disapproval (injunctive norms) and behavior (descriptive norms) of relevant socials referents. Injunctive norms are all about what you think others expect of you in a particular social context (e.g., “People important to me would approve of me protecting children from sexual violence”). An example of descriptive norms would then read “Most people like me will protect children from sexual violence” (Fishbein and Ajzen, 2010).

Perceived behavioral control is a person’s perception of their capacity and autonomy over their behavior, where capacity entails the level of confidence a person has in being capable to perform a behavior (e.g., “I am confident that, if I want to, I can protect children from sexual violence”). Autonomy refers to people’s belief that they have control over the behavior and that performing the behavior is up to them (e.g., “It is me who decides whether I protect children from sexual violence”; Fishbein and Ajzen, 2010).

Because educational programs cannot directly change behavior but can change behavioral determinants, understanding which determinants primarily influence a given behavior in a given population is an important first step in developing behavior change interventions (Crutzen et al., 2017). Besides establishing which determinants are the most promising targets for an intervention, it is important to identify critical subdeterminants, i.e., determining underlying beliefs. Thus, if perceived norms play a prominent role, it is vital to learn which social referents matter the most (i.e., peers, family, or other persons or groups). If behavioral control is the problem, we need to identify the perceived barriers or the (sub)skills people feel they lack.

The study presented below focuses on youth sport coaches as key players in the prevention of sexual violence toward child athletes in local sport settings. Almost all athletes begin their sport careers in local clubs at an early age, making safe sport environments crucial for their wellbeing and development. Since coaches play such a prominent role in youth sport, they are necessarily critical in their protection from harassment and abuse, most particularly sexual violence. We therefore conducted a survey among youth sport coaches to systematically identify the most relevant determinants of positive bystander behavior for future inclusion in a coach-bystander training program. The research question guiding this study is: what are the most relevant determinants of positive bystander behavior in the prevention of sexual violence in sport?

## Materials and methods

### Participants

This study used a cross-sectional survey design with a self-selection convenience sample of currently active adult youth sport coaches in Flanders, Belgium. After providing their informed consent (see Procedure), 1,741 participants aged ≥ 18 years started the online survey; 323 were excluded as they did not meet the inclusion criterion of having been actively involved as a youth sport coach in the past 12 months. Meeting the inclusion criteria, the remaining 1,422 participants completed the full survey. The average age of the participants was 36.7 years (*SD =* 14.06, range 18–79), the majority were men (*n* = 883, 63.7%). The participants coached mostly in soccer (*n =* 447, 28.1%), athletics/track and field (*n =* 271, 17%) and gymnastics (*n =* 115, 9.7%). In total 42 different sports were mentioned. Most of the participants worked as a volunteer (*n =* 1,150, 80.9%), had a coach qualification (*n* = 865, 60.8%), were members of a sports club with both recreational and competitive divisions (*n =* 979, 68.8%), and had more than 1 year experience as a coach (*n* = 1,238, 93.4%). There was an equal distribution in the age groups they coached (athletes aged below 12 years: *n =* 549, 38.6%; 12–18 years: *n =* 321, 22.6%; both groups: *n* = 552, 38.8%). All sociodemographic characteristics and details of the coaching context are summarized in Table 1.

**Table 1:** Sociodemographics of the coach-participants and coaching context.

Characteristics	*n*	*%*
Gender		
Male	883	62.1
Female	490	34.5
Unknown	49	3.4
Age of athletes coached		
Younger than 12 years old	549	38.4
Between 12 and 18 years old	321	22.6
Both groups	552	38.8
Athletes with disability		
Yes	105	7.6
No	1,278	92.4
Coaching context		
Recreational sport only	109	7.7
Competitive sport only	294	20.7
Both	979	68.8
Competition level athletes/clubs coached		
Local level	600	42.2
Regional level	721	50.7
National level	497	35.0
International level	99	7.0
Coaching status		
Volunteer	1,150	80.9
Employed or self-employed	176	12.4
Coaching qualification		
Yes	865	60.8
No	457	32.1

### Measures

Our group developed the online questionnaire “And what would you do?” specifically for our coach survey into their notions on bystander behavior with respect to sexual violence. As stated, we used the RAA (Fishbein and Ajzen, 2010) as a theoretical framework and the items were formulated following the RAA CIBERlite items (Crutzen and Peters, 2022). We divided the questionnaire into five parts with a total of 87 items (see Supplementary Table 1). First, three questions probed the coaches’ personal experiences with active bystander behaviors in response to incidents of sexual violence during their coaching activities in the last 12 months.

**Figure 1:**
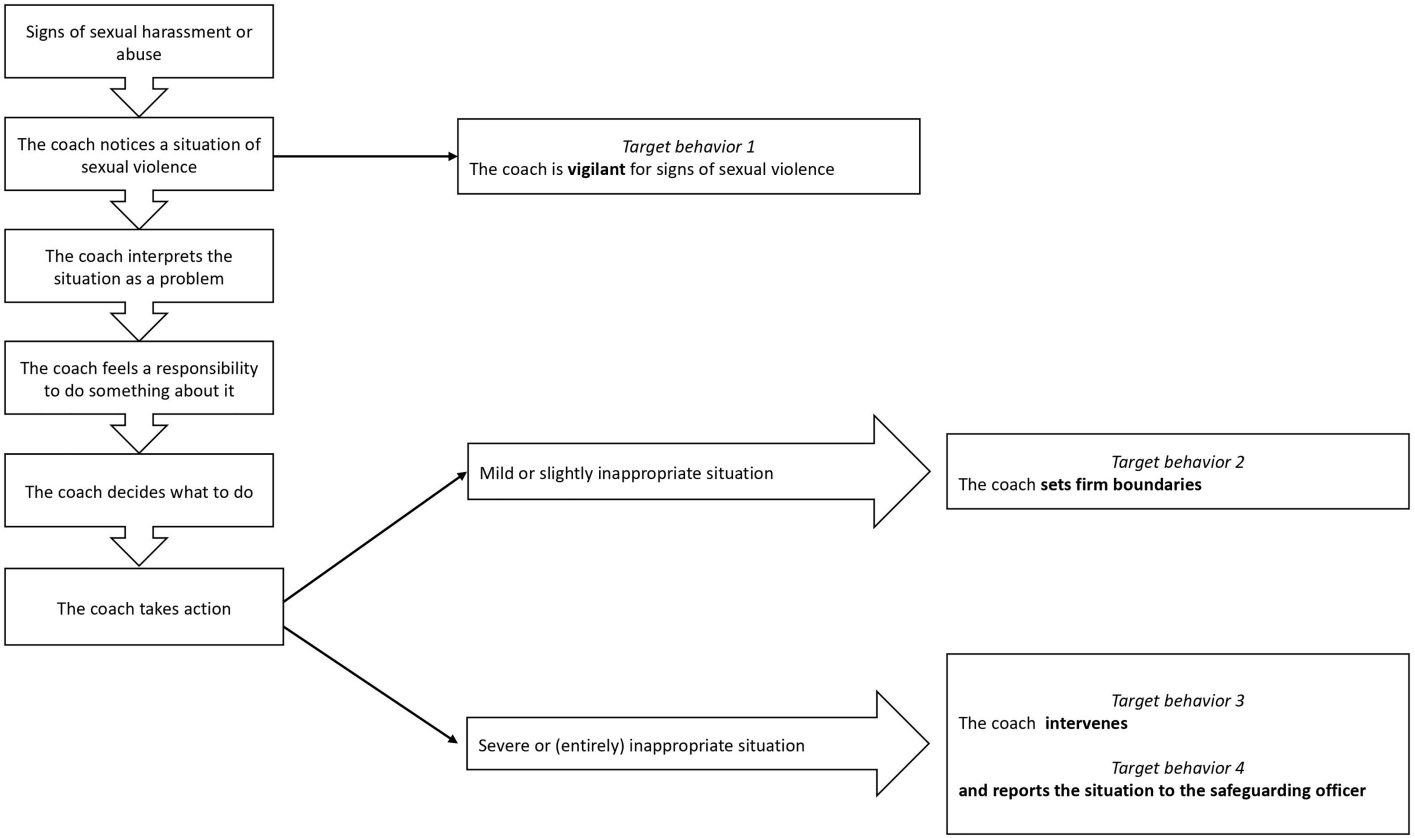
Overview coach bystander steps and their target behavior.

The following section assesses the subdeterminants of four target behaviors. Each target behavior is assessed in a dedicated section of the questionnaire. The four target behaviors were determined by applying the steps of bystander actions to the context of sport. The questionnaire focusses on the behavioral processes (i.e., the coach notes a situation, and the coach takes action), and not on the thought processes (e.g., the coach feels responsible to do something, makes a decision what to do). Regardless of the severity of an at-risk situation, the first two target behaviors are: (1) The coach is vigilant for signs of sexual violence, and (2) The coach sets firm boundaries in case of (signs of imminent) sexual violence. Depending on the level of severity, different follow-up actions might be required: (3) The coach intervenes in case of an incident of sexual violence, (4) The coach reports the incident to the club’s safeguarding officer. In order to present real-life scenarios, the questionnaire included two case descriptions with varying severity: one describing an incident including verbal sexual harassment, and one on sexual assault in the sports club. The severity of the actions required were determined following the guidelines in the Flag System, a pedagogical tool for sport stakeholders to adequately identify and react in situations of sexual violence (Van Haastrecht et al., 2017). Mild or slightly inappropriate behavior requires increased attention and setting firm boundaries, while more severe (contact) sexual behavior calls for a more profound intervention (e.g., end the inappropriate behavior, reporting to and involving the safeguarding officer; see Figure 1).

The first item of each target behavior assesses behavioral intent (e.g., *“Being a coach, it is my* intention *to be vigilant for signs of sexual abusive behavior,” “If I find myself in such a situation, it is my intention to take action”*) while the subsequent items test the determinants (i.e., attitude, perceived norms, and perceived behavioral control) of the bystander behavior in question.

In accordance with the RAA, we gauged instrumental and experiential attitude, both operationalized as (i) “belief expectation” (for instance, for instrumental attitude: *“Thinks that reporting to the safeguarding officer will improve the situation,”* and for experiential attitude: *“I feel comfortable setting firm boundaries in situation of sexual violence” and “I feel determined when I intervene in such a situation”*) and (ii) as ‘belief evaluation’ (*“I believe it is important to report an incident of sexual violence to a* safeguarding *officer”* and *“I need to feel comfortable when intervening,”* respectively).

Secondly, perceived norm was operationalized as injunctive norms and descriptive norms, with the first being divided into “motivation to comply” (e.g., *“I like to act the way fellow coaches would want me to act”*) and “perceived referent approval” (e.g., *“The club board will approve of my intervening in an incident involving sexual violence”*). Descriptive norms were assessed as “perceived referent behavior” (e.g., *“Most people like me would set boundaries in such a situation”*) and “identification with referent” (e.g., *“I want to do what fellow coaches would do when it comes to reporting the case to a* safeguarding *officer”*).

Finally, perceived behavioral control was assessed in terms of “autonomy” and “capacity,” with autonomy being further divided into “power of condition” (e.g.,*“Whether I intervene in a situation is completely up to me”*), “presence of condition” (e.g., *“My club has drawn up a code of conduct”*) and capacity into “subskill importance” (e.g., *“I think the ability to explain what is wrong and what is appropriate behavior is important”*) and “subskill presence” (e.g., *“I feel confident about being able to set boundaries”*).

Depending on the scale anchors, some subdeterminants were rated on a bidimensional seven-point scale and others with a unidimensional 5-point scale, but always with question-specific anchors (cf. Gehlbach and Brinkworth, 2011).

### Procedure

After obtaining ethical clearance from the Ethics Committee at the lead researcher’s institution (G-2020 122,035), the questionnaire was pre-tested by the project’s steering group consisting of child safeguarding in sport officers and practice developers, public health researchers, survivors of childhood sexual violence in sport, and representatives from sport organizations and authorities.

After the pre-test the additional adjustments were made, eligible sport coaches were recruited. A self-selection convenience recruitment strategy was applied, using the professional networks of partner organizations including the Flemish School for Coach Education, the Center Ethics in Sport, and several Flemish sport federations. All the partners distributed the call for participation, including a direct link to the questionnaire (www.enwatzoujijdoen.be), through their social media and information channels. The questionnaire was launched in June 2020 and data were collected from 2020-06-29 up to 2020-08-11. Upon accessing the questionnaire, participants were briefed about the survey objectives, informed that no personal data would be collected, and asked for their informed consent.

### Statistical analyses

All subdeterminants were recoded into a scale ranging from 0 to 1 to facilitate visual comparison, with the percentages of maximal possible method (POMP) generating raw maximum scores (Cohen et al., 1999). Given the problems with regression analyses for determinant selection (Crutzen and Peters, 2021), the relevant importance of the determinants of the coach-bystander behaviors tested was analyzed using confidence interval-based estimation of relevance (CIBER) technique. CIBER has been developed as a tool to select the most relevant (sub-)determinants of a behavior to inform the focus of behavioral change intervention (Crutzen et al., 2017). The technique visualizes the mean of each subdeterminant and the associations with the behavioral outcomes depicted in two panels, where the left-hand panel shows each variable’s distribution using raw data, as well as 99.99% confidence intervals (CI) for the means (i.e., how low or high participants score on the scale) presented as diamonds along the continuum of possible scores. The fill color of the diamonds gives an indication of the item means, with the color green representing higher means, blue means close to the scale center, and red lower means. The right-hand panel presents the correlation coefficients of the subdeterminants and the specified target (e.g., the behavior measure) with a 95% CI. Here the diamonds’ color reflects the association’s strength and direction, with green indicating a strong positive association. The grayer the diamonds are, the weaker the association, where a red diamond signifies a strong, negative association. Combined, the panels show associations with behavior and the degree of room for improvement. The CIBER plots were created using the R package *behavior change.*

Since with CIBER we seek to identify the most relevant (sub-)determinants of positive coach-bystander behaviors, we look for those factors that show either low-to-average means and a positive association with the target behavior, or those with average-to-high means and a negative association (Crutzen et al., 2017). Next, we calculated the potential for change index (PCI or PΔ) using SPSS (version 28), which quantitatively combines the room for improvement with the association with behavior (e.g., the product of (1) the difference between the (sub-)determinant’s mean and the scale maximum and (2) the squared correlation with intention). In other words, the PCI provides a quantitative summary of the information shown in the CIBER plots (Knittle and Peters, 2019). A threshold PCI value of ≥0.05 were taken to indicate (sub-)determinants relevant for inclusion in the coach-bystander intervention based on the patterns observed in the CIBER plots in combination with the corresponding PCI (note that this threshold represents our decision process in the context of this study and does not represent a heuristic meant for general application despite its coincidental similarity to the common default alpha). The PCI can conveniently aggregate much information into one quantitative metric, but because that inevitably also obfuscates potentially important information, we combined PCI and CIBER plots.

## Results

The majority of the coach-participants reported not to have noted or witnessed any case of sexual violence in their sports club in the previous 12 months (*n* = 1,184, 90.3%), while 78 (5.5%) had witnessed one such event, of whom 52 (*n* = 52/78; 66.7%) had intervened. Another 49 coaches (3.4%) reported to have witnessed several situations involving sexual violence, with six (*n* = 6/49; 12.2%) coaches not taking any action and nine (*n* = 9/49; 18.4%) having only rarely done so; 11 (*n* = 11/49; 22.4%) had acted on some occasions, 10 (*n* = 10/49; 20.4%) had intervened in most instances, while 13 (*n* = 13/49; 26.5%) coaches had intervened in each event. Most common reasons for not intervening were a failure to (correctly) identify the behavior as sexual violence (“*it looked harmless*”), the lack of knowledge and skills to name and/or address the issue, and an inaction due to the perpetrator’s hierarchical position.

**Figure 2:**
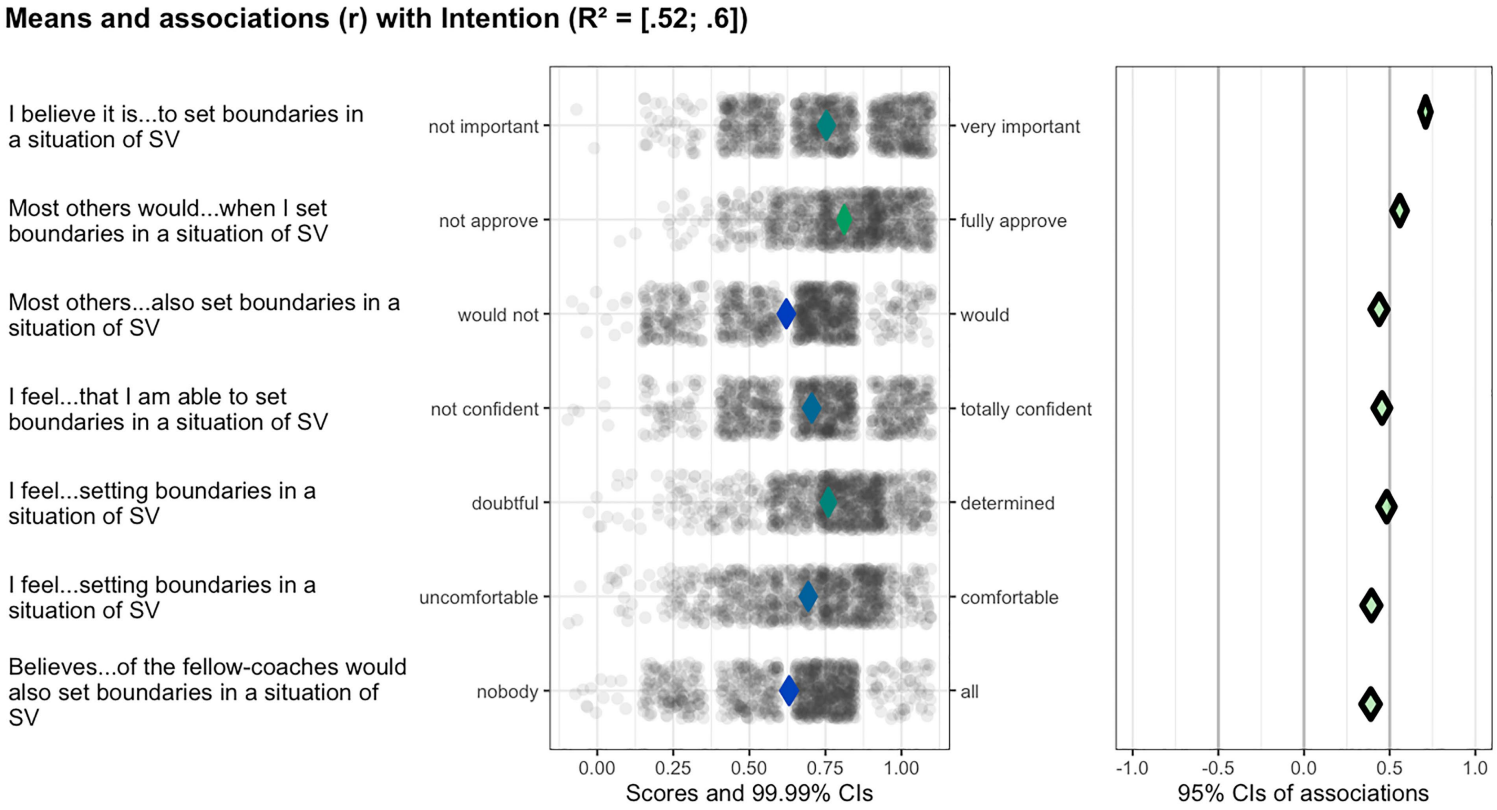
Ciberplot target behavior 2: the coach sets firm boundaries in case of an incident of sexual violence.

**Figure 3:**
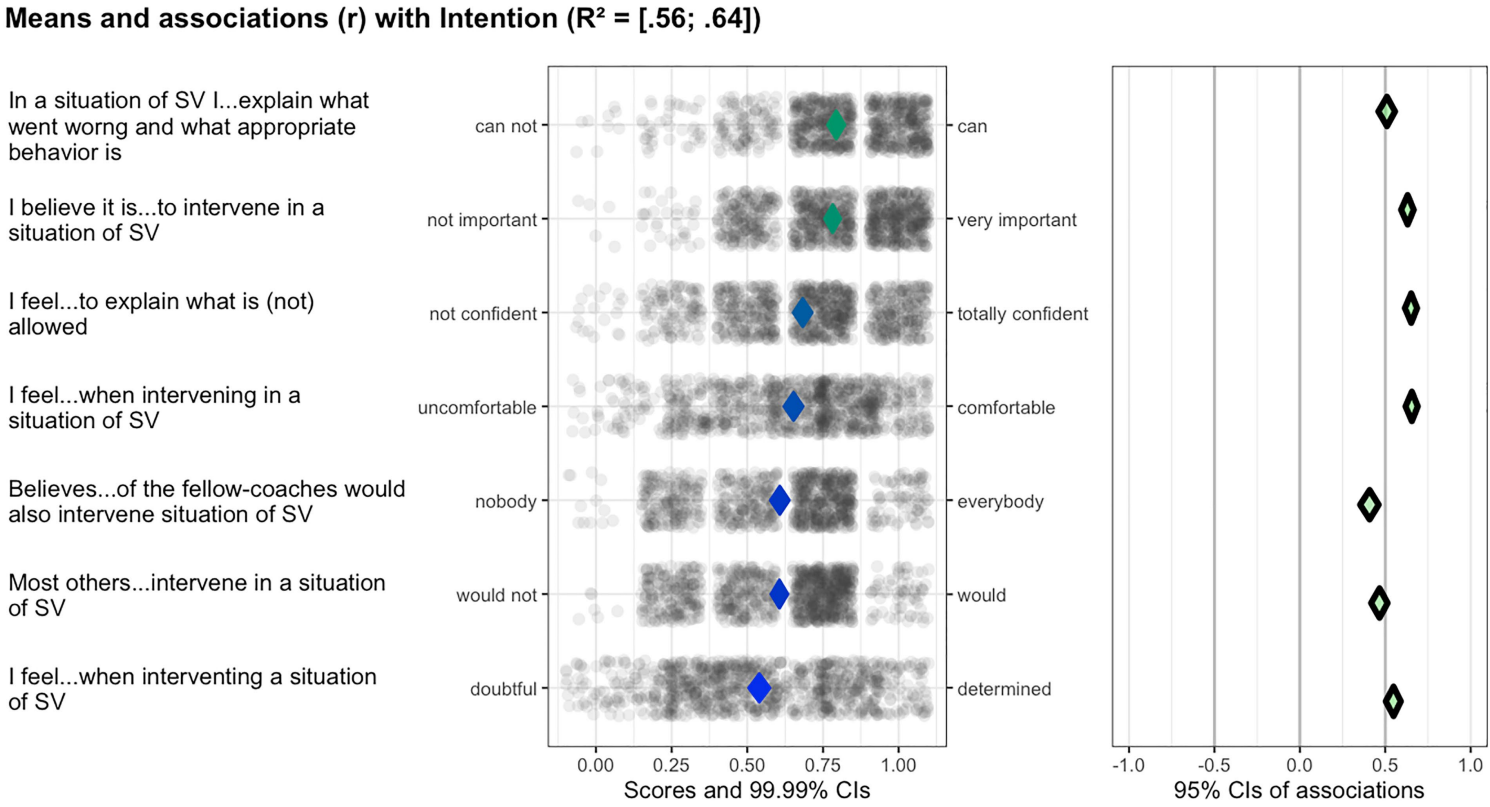
Ciberplot target behavior 3: the coach intervenes in a situation of sexual violence.

**Figure 4:**
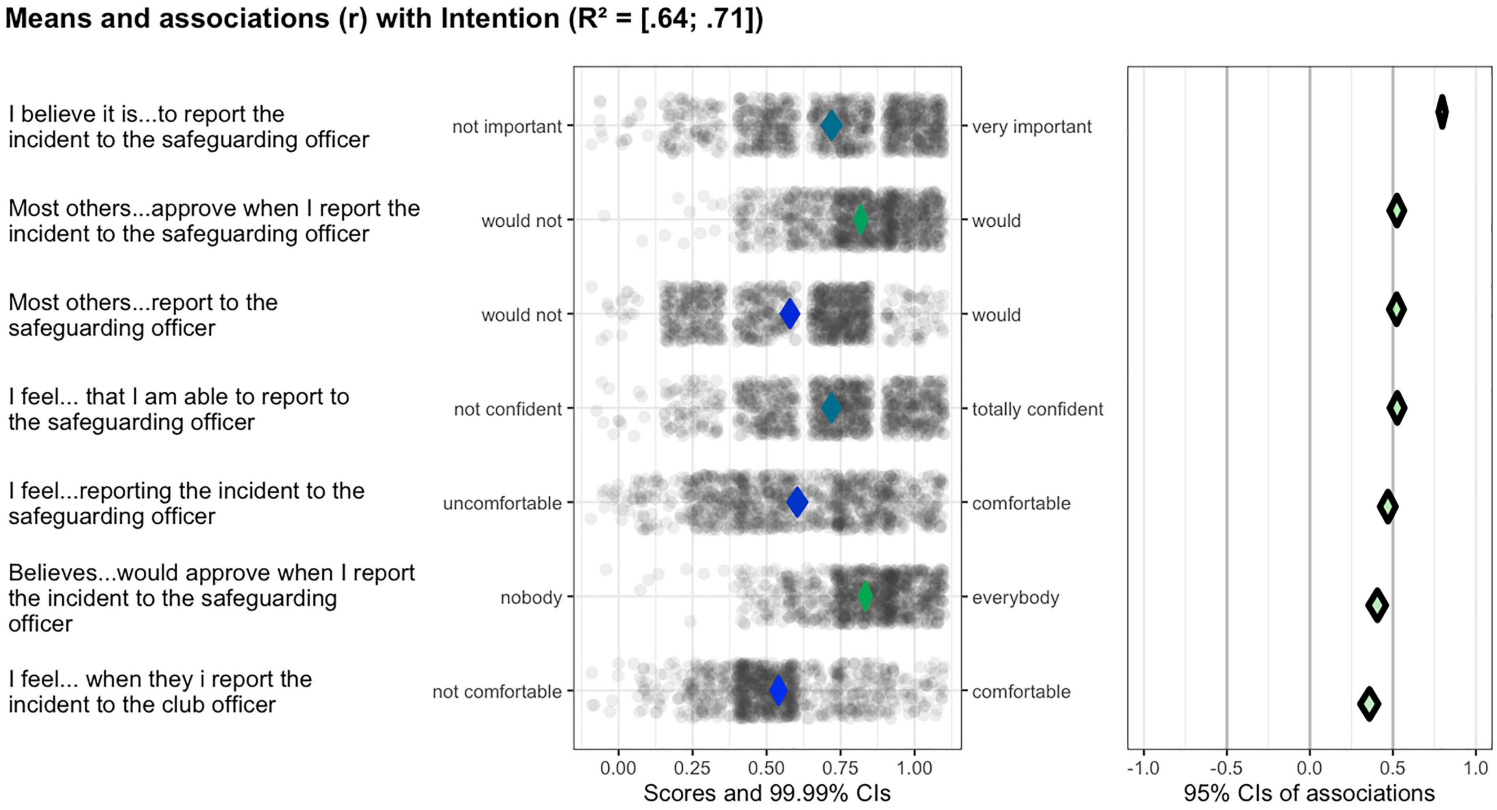
Ciberplot target behavior: the coach reports the situation to the safeguarding officer.

Next, we will describe the (sub-)determinants our analyses identified as the most relevant for the promotion of positive coach-bystander behavior. Figures 2–4 provide examples of the analysis process for several of these target behaviors.

### Target behavior 1: The coach is vigilant for signs of sexual violence

The intention of coaches to be vigilant for signals of sexual violence was high (*M* = 0.92, *SD* = 0.15). Inspection of the CIBER plots and PCIs suggested that only one determinant is relevant for this target behavior: instrumental attitude belief evaluation (*M* = 0.87, *SD* = 0.18, r = 0.61, PΔ = 0.05; see Table 2). This may seem surprising given the high mean but is explained by the high correlation coefficient. Perceived referent behavior (*M* = 0.66, *SD* = 0.20, r = 0.29 PΔ = 0.03) and subskill presence (*M* = 0.60, *SD = 0*.21, r = 0.27, PΔ = 0.03) were also positively associated with intention, with the determinants’ mean scores falling in the middle of the scale. However, both the corresponding correlation coefficients and PCIs were low, suggesting that these determinants show little promise as intervention targets. The sample mean of the determinant perceived referent approval (*M* = 0.83, *SD* = 0.18, r = 0.36, PΔ = 0.02) was already high, indicating there is little room for improvement, while the correlation coefficient was moderate, meaning that, as a result, the potential for change is low. Similarly, the associations with intention and the PCIs of the remaining determinants of this target behavior were too weak to warrant attention in an intervention.

**Table 2:** Target behavior 1: the coach is vigilant for signs of sexual violence.

Determinants	Subdeterminants	*P*Δ	Correlation (r)	Means (SD)
Instrumental attitude belief evaluation	*Believes vigilance to be important*	0.05	0.61	0.87 (0.18)
Perceived referent behavior	*Believes others would be vigilance*	0.03	0.29	0.66 (0.20)
Subskill presence	*Feels confident about detecting abilities*	0.03	0.27	0.60 (0.21)
Perceived referent approval	*Thinks others approve of his/her being vigilant*	0.02	0.36	0.83 (0.18)
Experiential attitude belief expectation	*Feels comfortable being vigilant*	0.02	0.25	0.66 (0.21)
Power of condition	*Being vigilant is own decision*	0.01	0.22	0.80 (0.19)

All subdeterminants were recoded to a 0–1 scale.

### Target behavior 2: The coach sets firm boundaries in case of an incident of sexual violence

The intention of coach-participants to set firm boundaries was high (*M* = 0.86, *SD* = 0.18). In total seven determinants were indicated as relevant for inclusion in the coach-bystander intervention. The first group includes subskill presence (*M =* 0.71*, SD = 0*.22, r = 0.46, PΔ = 0.06), two items regarding experiential attitude belief expectation (*M* = 0.76, *SD* = 0.20, r = 0.48, PΔ = 0.06; *M* = 0.69, *SD* = 0.22, r = 0.39, PΔ = 0.05), and two items on perceived referent behavior (*M* = 0.62, *SD* = 0.22, r = 0.44, PΔ = 0.07; *M* = 0.63, *SD* = 0.22, r = 0.39, PΔ = 0.06). They were all seven strongly associated with this target behavior (see Table 3; Figure 2). Given their mid-scale means and moderate associations with intention, these determinants exceeded the PCI threshold (0.05) and are thus relevant for a coach-bystander intervention, which also holds for the following determinants: perceived referent approval (*M = 0.*81, *SD* = 0.17, r = 0.56, PΔ = 0.06) and instrumental attitude belief evaluation (*M* = 0.75, *SD = 0*.14, r = 0.71, PΔ = 0.12) given their high correlation coefficients manifesting in high PCIs. The other determinants of this target behavior all had relatively high sample means. One group (instrumental attitude belief expectation, belief evaluation, subskill presence, and perceived referent approval) had a low association with intention (*r* ≤ 0.40), rendering these determinants immaterial since they will not induce behavior change. Although the remaining determinants had high associations with intention (*r* ≥ 0.40), they showed little room for improvement, as was reflected by their low PCI values.

**Table 3:** Target behavior 2: the coach sets firm boundaries in case of an incident of sexual violence.

Determinants	Subdeterminants	*P*Δ	Correlation (r)	Means (SD)
Instrumental attitude belief evaluation	*Believes setting clear boundaries is important*	0.12	0.71	0.75 (0.14)
Perceived referent behavior	*Believes others would also set boundaries*	0.07	0.44	0.62 (0.22)
Perceived referent approval	*Thinks others would approve of his/her setting boundaries*	0.06	0.56	0.81 (0.17)
Subskill presence	*Feels confident about being able to set boundaries*	0.06	0.46	0.71 (0.22)
Experiential attitude belief expectation	*Feels determined when it comes to setting boundaries*	0.06	0.48	0.76 (0.20)
Perceived referent behavior	*Believes fellow coaches would also set boundaries*	0.06	0.39	0.63 (0.22)
Experiential attitude belief expectation	*Feels comfortable setting boundaries*	0.05	0.39	0.69 (0.22)
Instrumental attitude belief expectation	*Believes setting boundaries will help improve the situation*	0.04	0.42	0.75 (0.22)
Instrumental attitude belief evaluation	*Believes improving the situation is important*	0.03	0.50	0.90 (0.13)
Subskill presence	*He/she is able to explain what is (not) allowed*	0.03	0.42	0.82 (0.19)
Perceived referent approval	*Finds approval from fellow trainers for setting boundaries important*	0.03	0.41	0.81 (0.17)
Importance subskill	*Finds being able to explain what is (not) allowed important*	0.02	0.32	0.81 (0.19)
Identification with referent	*Likes to act like fellow coaches regarding boundaries*	0.02	0.23	0.58 (0.29)
Perceived referent approval	*Thinks the club board would approve of his/her setting boundaries*	0.02	0.36	0.86 (0.15)
Power of condition	*Setting boundaries is own decision*	0.01	0.32	0.87 (0.17)
Motivation to comply	*Likes to act the way fellow coaches would want him/her to act*	0.01	0.15	0.55 (0.28)
Power of condition	*Finds the club having a code of conduct important*	0.01	0.21	0.75 (0.25)
Presence of condition	*Thinks the club probably has a code of conduct*	0.01	0.16	0.73 (0.29)
Motivation to comply	*Likes to act the way the club board wants him/her to act*	0.01	0.14	0.60 (0.28)

All subdeterminants were recoded to a 0–1 scale.

### Target behavior 3: The coach intervenes in a situation of sexual violence

The overall intention was high (*M* = 0.82, *SD* = 0.21) and seven determinants of this target behavior were identified as eligible for inclusion in the intervention (see Table 4; Figure 3): instrumental attitude belief evaluation (*M* = 0.78, *SD* = 0.22, r = 0.63, PΔ = 0.09), two items regarding experiential attitude belief expectation (*M* = 0.65, *SD* = 0.25, r = 0.66, PΔ = 0.15; *M* = 0.54, *SD = 0*.28, r = 0.55, PΔ = 0.14), and two items regarding subskill presence (*M* = 0.68, *SD = 0*.25, r = 0.65 PΔ = 0.14; *M* = 0.79, *SD* = 0.22, r = 0.51, PΔ = 0.05). Two items of perceived referent behavior (*M* = 0.61, *SD = 0*.23, r = 0.41, PΔ = 0.07; *M* = 0.61, *SD = 0*.24, r = 0.34, PΔ = 0.05) had a moderate correlation coefficient, with the PCI score meeting the threshold. The associations with intention and the PCIs of the remaining determinants of this target behavior were too weak, excluding them as targets for the intervention.

**Table 4:** Target behavior 3: the coach intervenes in a case of a situation of sexual violence.

Determinants	Subdeterminants	*P*Δ	Correlation (r)	Means (SD)
Experiential attitude belief expectation	*Feels determined when intervening*	0.15	0.66	0.65 (0.25)
Experiential attitude belief expectation	*Feels comfortable intervening*	0.14	0.55	0.54 (0.28)
Subskill presence	*Feels confident in his/her ability to intervene*	0.14	0.65	0.68 (0.25)
Instrumental attitude belief evaluation	*Believes intervening to be important*	0.09	0.63	0.78 (0.22)
Perceived referent behavior	*Believes fellow trainers would also intervene*	0.07	0.41	0.61 (0.23)
Subskill presence	*He/she is able to explain what went wrong and what appropriate behavior is*	0.05	0.51	0.79 (0.22)
Perceived referent behavior	*Believes others would also intervene*	0.05	0.34	0.61 (0.24)
Perceived referent approval	*Thinks important others would approve of his/her intervening*	0.03	0.43	0.83 (16)
Perceived referent approval	*Thinks fellow trainers would approve of his/her intervening*	0.03	0.34	0.77 (0.17)
Identification with referent	*Likes to be like fellow coaches regarding intervening*	0.02	0.21	0.56 (0.29)
Motivation to comply	*Likes to act the way fellow coaches would want him/her to act*	0.02	0.19	0.50 (0.28)
Presence condition	*Thinks the club probably has a code of conduct*	0.02	0.25	0.74 (0.29)
Power of condition	*The decision to intervene is entirely up to him/her*	0.02	0.34	0.87 (0.17)
Perceived referent approval	*Thinks the club board would approve of his/her intervening*	0.01	0.29	0.83 (0.16)
Experiential attitude belief evaluation	*Thinks having a sense of determination is important*	0.01	0.18	0.68 (0.22)
Instrumental attitude belief expectation	*Thinks intervening will help improve the situation*	0.01	0.18	0.72 (0.17)
Motivation to comply	*Likes to act the way the club board would want him/her to act*	0.01	0.14	0.57 (0.27)
Importance subskill	*Thinks the ability to explain what was wrong and what appropriate behavior is very important*	0.01	0.18	0.82 (0.20)
Power of condition	*Thinks there needs to be a code of conduct explaining when to intervene*	0.01	0.14	0.78 (0.23)
Experiential attitude belief evaluation	*Thinks feeling comfortable to intervene is important*	0.00	0.02	0.60 (0.25)

All subdeterminants were recoded to a 0–1 scale.

### Target behavior 4: The coach reports the situation to the safeguarding officer

Intention was high (*M* = 0.78, *SD* = 0.24), with nine determinants appearing relevant for the intervention (see Table 5; Figure 4): instrumental attitude belief evaluation (*M* = 0.72, *SD* = 0.27, r = 0.80, PΔ = 0.18), perceived referent behavior (*M* = 0.58, *SD* = 0.24, r = 0.52, PΔ = 0.12; *M* = 0.57, *SD* = 0.25, r = 0.49 PΔ = 0.10), subskill presence (*M* = 0.72, *SD* = 0.24, r = 0.53, PΔ = 0.08), instrumental attitude belief expectation (*M* = 0.73, *SD* = 0.18, r = 0.43, PΔ = 0.05), two aspects of experiential attitude belief expectation (*M* = 0.60, *SD* = 0.27, r = 0.47, PΔ = 0.09; *M* = 0.54, *SD = 0*.21, r = 0.36, PΔ = 0.06), two regarding perceived referent approval (*M* = 0.82, *SD* = 0.17, r = 0.53, PΔ = 0.05; *M* = 0.77, *SD* = 0.18, r = 0.44, PΔ = 0.05), and two regarding perceived referent behavior (*M* = 0.58, *SD* = 0.24, r = 0.52, PΔ = 0.12; *M* = 0.57, *SD* = 0.25, r = 0.49, PΔ = 0.10). All these determinants showed moderate-to-high correlations and PCIs ≥ 0.05. The associations with intention and the PCIs of the remaining determinants of this target behavior were too weak and are thus not considered for the intervention.

**Table 5:** Target behavior 4: the coach reports the incident to the safeguarding officer.

Determinants	Subdeterminants	*P*Δ	Correlation (r)	Means (SD)
Instrumental attitude belief evaluation	*Believes reporting is very important*	0.18	0.80	0.72 (0.27)
Perceived referent behavior	*Believes others would also report*	0.12	0.52	0.58 (0.24)
Perceived referent behavior	*Believes fellow coaches would also report*	0.10	0.49	0.57 (0.25)
Experiential attitude belief expectation	*Feels comfortable about reporting*	0.09	0.47	0.60 (0.27)
Subskill presence	*Feels confident that he/she would report*	0.08	0.53	0.72 (0.24)
Experiential attitude belief expectation	*Feels loyal to fellow trainers when reporting*	0.06	0.36	0.54 (0.21)
Perceived referent approval	*Thinks important others would approve of his/her reporting*	0.05	0.53	0.82 (0.17)
Instrumental attitude belief expectation	*Thinks reporting will improve the situation*	0.05	0.43	0.73 (0.18)
Perceived referent approval	*Thinks fellow coaches would approve of his/her reporting*	0.05	0.44	0.77 (0.18)
Identification with referent	*Likes to be like fellow trainers when it comes to reporting*	0.03	0.27	0.56 (0.28)
Motivation to comply	*Likes to act the way fellow coaches would want him/her to act*	0.03	0.25	0.51 (0.28)
Motivation to comply	*Likes to act the way the club board want him/her to act*	0.03	0.26	0.57 (0.27)
Perceived referent approval	*Thinks the club board would approve of his/her reporting*	0.03	0.41	0.83 (0.16)
Subskill presence	*He/she is able to report any situation discreetly*	0.02	0.37	0.86 (0.18)
Power of condition	*Deciding to report is own decision*	0.01	0.30	0.87 (0.17)
Instrumental attitude belief evaluation	*Believes improving the situation is important*	0.01	0.28	0.84 (0.19)
Presence condition	*Thinks it is important to have a safeguarding officer to facilitate reporting*	0.01	0.20	0.78 (0.23)
Power of condition	*The presence of a safeguarding officer facilitates reporting*	0.01	0.15	0.76 (0.32)
Experiential attitude belief evaluation Importance subskill	*Thinks maintaining a sense of loyalty is important* *Feels being discreet when reporting is crucial*	0.00 0.00	0.09 0.06	0.59 (0.26) 0.83 (0.20)

All subdeterminants were recoded to a 0–1 scale.

In total, 24 determinants were identified as eligible for inclusion in the intervention. Only one applied to *vigilance,* seven to both *setting firm boundaries* and to *intervening*, and most of the determinants, nine, related to the fourth target behavior *reporting to the safeguarding officer.* In total, 46% of these were related to attitude, 37% to perceived norms, and 17% to perceived behavioral control. Experiential attitude in terms of belief expectation, perceived referent behavior, and instrumental attitude in terms of belief evaluation, as well as perceived referent approval were most pertinently present.

## Discussion

We adopted the CIBER approach to evaluate determinants of positive coach-bystander behaviors to develop a bystander intervention and therefore help to prevent sexual violence in youth sport. The majority (90.3%) of the 1,422 coaches responding to our online survey reported not to have witnessed any incident involving sexual violence in the last 12 months. This could mean that there were no actual incidents or that coaches may not have been successful in detecting (signs of) sexual violence (Mountjoy et al., 2016; Vertommen et al., 2016a), which in turn could be explained by the fact that coaches do not have the knowledge to correctly identify and assess a situation of sexual violence. Additionally, due to the COVID-19 lockdown that coincided with a large part of the study period, most organized sport activities were cancelled and therefor the opportunities to witness any incidents may have been relatively scarce. Nevertheless, the fact that 10% did report witnessing one or more incidents in the given timeframe highlights the importance of raising awareness and educating coaches about sexual violence in sport.

By combining CIBER plots with the potential for change we were able to identify the most relevant factors contributing to the four coach-bystander behaviors that could be targeted in sexual-violence prevention programs for coaches. Reassuringly, we found high intention for all four target behaviors, reflecting that the coaches in our sample would be inclined to intervene when witnessing sexual violence during their watch, with the majority of those having observed an actual incident following up on this intent by taking action. Still, some did not intervene.

In line with previous studies (Banyard et al., 2007; Jouriles et al., 2018) attitude was found to be an important and changeable determinant in bystander intervention. In our study, the experiential attitude in terms of belief expectation and instrumental attitude in terms of belief evaluation are put forward as promising determinants to focus on in the development of coach-bystander interventions. The same applies for perceived norms, which has been pointed out in the literature as an important determinant (e.g., Brown and Messman-Moore, 2010; McMahon, 2015; Santacrose et al., 2020), and was also confirmed in our results.

Of perceived behavioral control (i.e., perception of their capacity and autonomy on their behavior), subskill presence, played only a role in two target behaviors regarding taking action: setting firm boundaries and reporting to the safeguarding officer. Although various studies on topics ranging from child maltreatment to sexual violence show that higher levels of confidence are related to a greater willingness to offer help (e.g., Mudde et al., 2007; Moynihan and Banyard, 2008), our study does not confirm this. Perceived behavioral control proved the least relevant attribute to target and therefor this determinant is not considered to as a key element to target when developing a coach-bystander intervention.

Like Witte et al. (2017), who observed that many bystanders who had intervened in a violent situation reported their actions had made them feel positive, proud and empowered (with far fewer reporting negative sentiments), we found in our study that all but one of the items gauging the participants’ expectations about their behaviors, and most specifically their experiential attitude on belief expectations (i.e., affective consequences), were selected and thus core targets for a prevention program. We wish to note that the subdeterminant “*As a coach I feel comfortable being vigilant for signs of sexual abuse,*” not approached the threshold score (0.05) but may still be relevant as an intervention target.

Of perceived referent behavior, the second most relevant factor we identified, only the (sub-)determinants of the target behavior on being vigilant were not relevant. All other items (e.g., “*Most people like me will set boundaries in case of sexual violence”* and “*Most people like me would intervene in a situation of sexual violence”*) appeared important for fostering positive bystander behavior. Levine et al. (2019) earlier highlighted the importance of utilizing shared group membership through which intervening becomes the ‘social’ norm among peers when threatening or violent events are being witnessed. A sexual-violence prevention program for coaches of local sport clubs should then focus on this sense of group membership and prevailing (pro)active peer behavior.

Looking at instrumental attitude, we found belief evaluation (i.e., how one rates the value of a particular behavior; Fishbein and Ajzen, 2010) to be relevant for all four target behaviors—and the only determinant that was significant for vigilance (target behavior 1). This latter would suggest that a prevention program should also be aimed at reinforcing coaches’ willingness to be watchful for (signs of) sexual violence.

Perceived referent approval (i.e., appreciation by relevant referents) was the last most important determinant found in our study, and was relevant for all subdeterminants of all three target behaviors, except for vigilance. Negative social group norms can hamper one’s intention to intervene as a bystander, which refers to the approval of others. In the context of sport this mainly refers to fellow-coaches and club managers. Based on our findings, not all coaches in this sample can count on the approval of their fellow trainers and managers when intervening in case of an incident of sexual violence.

Summing up, our study shows that the coaches in Flanders that responded to our survey already show high levels of intention to engage in the desired positive bystander behaviors we evaluated but that there is room for improvement. Our results will aid the development of a well-founded sexual violence prevention program for youth sport coaches. Methods and applications to effectuate positive attitudes (experiential attitude in terms of belief expectation and instrumental attitude in terms of belief evaluation) and positive perceived norms (perceived referent behavior and perceived referent approval) will be key in the development of effective intervention programs. Finally, although determining features of target behaviors can differ per population, region, and over time, the patterns we identified in the youth sport context may still prove useful for prevention professionals in other settings who lack the means to conduct determinant studies themselves (Fishbein and Ajzen, 2010).

### Limitations and future research

Several limitations warrant mentioning. Essentially, our study is restricted to active coaches and their role in preventing sexual violence in (youth) sports. Their views, reflected in these findings, do not necessarily apply to other members of the sport community that are also crucial stakeholders in the prevention of sexual violence. Secondly, while sexual violence appears to be correlated with other types of interpersonal violence toward child athletes (Vertommen et al., 2016b), this study is limited to sexual violence only. It is possible that influential determinants of bystander behavior in case of physical or psychological violence are different. The same applies for technology facilitated violence, for which the bystander behavioral determinants could be different. Thirdly, our questionnaire did not specifically focus on vulnerable athletes (e.g., sport participants with disabilities or those who identify as LGBTQI+). In order to gain specific information about coach bystander behaviors and determinants in case of those other forms of interpersonal violence, or in those specific target groups, additional research is required.

This study uses a self-selection convenience sample and touches on a sensitive topic. When interpreting the results, one should keep in mind the ‘sampling bias.’ It is possible that the current sample do not reflect the average target population (i.e., coaches in Flanders), as it is likely that only those coaches already convinced for the need for safeguarding and taking an interest in the prevention of sexual violence chose to complete our questionnaire (Lavrakas, 2008), which might then also explain why the intention for all targeted bystander behaviors was high. Arguably, our recruitment procedure may have excluded coaches with lower interest and less positive intentions.

The majority of participants reported not to have witnessed any incidents within the last 12 months. Since we used two hypothetical instances of sexual violence to explore coach-bystander behaviors, it is feasible that they never did, but it is also possible they did not report noteworthy events because they did not identify them as harmful. Speculatively, some coaches may even have seen signs or incidents but were reluctant to report this because they had failed to take action and, in hindsight, felt embarrassed. It is also likely that participants were reluctant to report these experiences because of social desirability (King, 2022).

Either way, their responses may not necessarily reflect true intentions or behaviors. As alluded to above, the COVID-19 measures may also have limited observations of harmful incidents. Since verification of actual behaviors poses marked problems, we need to rely on assessments of intentions to exhibit the desired (pro)active bystander behaviors, where intentions need not reflect actual behavior when this is called for.

In our analysis, we opted for CIBER since it has the advantage of combining the measures of room for improvement and association strengths. However, the quality of the output depends on the quality of the operationalizations, in our case the selection and formulation of the (sub-)determinants (Crutzen et al., 2017). Our survey is based on the RAA and in this phase, although it is one of the most widely utilized theories in the field of health promotion and behavior change, other models (e.g., social cognitive theory, social ecological model) may add to our findings and help further explain or identify more determining features of positive bystander behaviors. The cultural and organizational context of sport can be an important aspect to illuminate. For example, the ‘winning at all costs’ mentality and the hierarchical nature of the relationships that is present in sport, are part of this environment (Roberts et al., 2020). These environmental aspects may have additional implications when developing a coach-bystander intervention.

We feel that our survey analysis has yielded interesting leads for the development of an intervention for coaches aimed at preventing sexual violence in youth sport and, bearing in mind that the patterns we observed may differ, they may inform prevention strategies for other types of violence and in other contexts. We do recommend conducting a dedicated determinant study when the necessary resources are available. The focus of this research should then be on identifying bystander behavior in, among other areas of abuse (including technology facilitated violence), psychological or physical violence, and include all bystanders in the given setting. If we can develop programs to educate and train all actors in local or national sports, i.e., besides coaches the athletes themselves, their parents or partners, and club managers, and if we manage to procure endorsement by (inter)national umbrella organizations, we will be far better able to ensure that sports can be practiced in a safe environment.

## Conclusion

The CIBER approach in combination with the potential change index was applied to gain a better understanding of the predictors of positive coach-bystander behavior with respect to sexual violence and to subsequently select the determinants in order to develop a coach-bystander intervention. The results showed that for the prevention of sexual violence in sport, coach-bystander interventions should target specific (sub-)determinants of their attitude toward and perceived norms of the desired (pro)active behaviors. The aims of such interventions should focus in the first place to increase the positive affective consequences coaches’ experience when engaging in the target behaviors. When intervening in situation of sexual violence coaches must feel comfortable and determined in order to act as a prosocial bystander. Secondly, the sense of group membership among coaches and manager in the sport club needs to be reinforced. Intervening in case of an incident of sexual violence needs to be the social norm in the sport club. And lastly, the focus should lay on the evaluation of the value of the specific target behaviors. When intervening becomes the social norm, fellow-coaches and managers will give their approval when a coach intervenes in case of an incident of sexual violence. Although determining factors may vary for different types of abuse and sport settings, the outcomes presented can also inform other violence-in-sport prevention campaigns.

## Data availability statement

The raw data supporting the conclusions of this article will be made available by the authors, without undue reservation.

## Ethics statement

The studies involving human participants were reviewed and approved by Thomas More University Ethics Committee (G-2020 12 2035). The patients/participants provided their written informed consent to participate in this study.

## Author contributions

HV was involved in the conceptualization, funding acquisition, investigation, and methodology and wrote the original and final draft of the manuscript. TV was involved in the conceptualization, funding acquisition, investigation, methodology, and project administration and contributed to the revision of the original draft of the manuscript. G-JP was involved in the conceptualization, investigation, and methodology and contributed to the revision of the original draft of the manuscript. All authors contributed to the article and approved the submitted version.

## Funding

This project received internal funding from Thomas More Research (M-OHC-190086).

## Conflict of interest

The authors declare that the research was conducted in the absence of any commercial or financial relationships that could be construed as a potential conflict of interest.

## Publisher’s note

All claims expressed in this article are solely those of the authors and do not necessarily represent those of their affiliated organizations, or those of the publisher, the editors and the reviewers. Any product that may be evaluated in this article, or claim that may be made by its manufacturer, is not guaranteed or endorsed by the publisher.

## Supplementary materials

The Supplementary Materials for this article can be found online at: https://www.frontiersin.org/articles/10.3389/fpsyg.2022.862220/full#supplementary-material
