# Supplementary material for: Preventing sexual violence in sport: Determinants of positive coach-bystander behavior
Source: Front Psychol. 2022 Jul 20;13:862220. doi: 10.3389/fpsyg.2022.862220 (PMC9350517; doi:10.3389/fpsyg.2022.862220)
Supplement: Supplementary file 1 [file Table_1.DOCX]

**Questionnaire #AndWhatWouldYouDo?**

*This questionnaire is based on several theoretical applications:*

- **The Reasoned Action Approach (Fishbein & Ajzen, 2010):** It extensively described how to measure its components. These descriptions have been further specified in a set of Decentralized Construct Taxonomies and CIBERlite questions, which we have used in this questionnaire.
- **Decentralized Construct Taxonomies (DTCs):** DCTs were developed to enable unequivocal reference to specific constructs with their corresponding definitions and instructions for developing measurement instruments (and code measurement instruments, when doing a literature review; and elicit construct content and code the resulting data, when doing qualitative research). These DCTs enable unequivocal reference to these constructs without requiring central curation. You can read more about them at <https://r-packages.gitlab.io/psyverse/> and the 'Full' tab at <https://a-bc.gitlab.io/dct-raa/>. We have used the DCTs by first translating the different questions template as listed at <https://a-bc.gitlab.io/dct-raa/>.
- **CIBERlite:** Ciberlite questions are brief and calibrated direct measures for behavioral determinants bases on the RAA (<https://osf.io/2uwxp/wiki/home/>)

| Q1 | Briefing and informed consent | |
| --- | --- | --- |
| **Demographics and information about current coaching position** | | |
| Q2 | Where you active as a coach in the last 12 months in a sport club? | Yes  No |
| Q3 | Do you train children (younger than 18 year)? | Yes, younger than 12 years old  Yes, between 12 and 18 years old  Both groups  No |
| Q4 | What is your age? | *Dropdown 18 - 99* |
| Q5 | What is your gender? | Man  Woman  I am …  I prefer not to say |
| Q6 | Do you train children with disabilities? | Yes  No |
| Q7 | What is the coaching context of your sport club? | Recreational context  Competitive context  Both |
| Q8 | What is the competition level of your sport club? | Local level  Regional level  National level  International level |
| Q8 | What sports are practiced in your sport club? | *Dropdown* |
| Q9 | How many hours do you coach (hours/week) | *Open question* |
| Q10 | How many years of experience do you have as a coach? (in years) | *open question* |
| Q11 | What is your coaching status? | Volunteer  Employed or self-employed |
| **Response to incidence of sexual violence during coaching activities in the last 12 months** | | |
| Q12 | How many times did you witness a situation of sexual violence in the last 12 months? | Not once in the last 12 months  One situation  Several situations |
| Q13 | *In case of one situation:*  Did you take any action? | Yes  No |
| Q14 | *In case of several situation:*  How many times did you take action? | Never - Rarely - Sometimes - Most of the times - Always |
| Q15 | If you want, you can tell us more about why you did or did not something about the situation | *Open question* |
| ***Target behavior 1: The coach is vigilant for signs of sexual violence*** | | |
| Q16 | As a coach, I intend to be vigilant for signals of sexual violence. | Absolutely not - Probably not - Maybe - Probably - Absolutely |
| Q17 | For me, being vigilant for signs of sexual violence is … | Totally not important - Slightly important - Moderately important - Important - Very Important |
| Q18 | For me, being vigilant for signs of sexual violence is… | Very uncomfortable - Uncomfortable  Somewhat uncomfortable- Neither comfortable, nor uncomfortable- Somewhat comfortable - Comfortable - Very comfortable |
| Q19 | Most others whose opinions I value would… that I am being vigilant for signs of sexual violence. | Totally disapprove - Disapprove - Somewhat disapprove - Neither approve, nor disapprove - Somewhat approve  Approve - Totally approve |
| Q20 | How many people like you, would be vigilant for signal of sexual violence? | Nobody – Some people – About half of the people – Most people – Everybody |
| Q21 | I am … that if I want to, I can be vigilant for signals of sexual violence. | Not confident at all - Slightly confident  Moderately confident - Confident  Extremely confident |
| Q22 | Whether I am vigilant for signs of sexual violence, is… | Not up to me - Slightly up to me - Moderately up to me - Mainly up to me  Completely up to me |
| ***Target behavior 2: The coach sets firm boundaries in case of an incident of sexual violence*** | | |
| Q23 | I intend to set firm boundaries in case of an incident of sexual violence | Absolutely not - Probably not - Maybe - Probably - Absolutely |
| Q24 | For me, setting firm boundaries in case of an incident of sexual violence is … | Totally not important - Slightly important - Moderately important - Important - Very Important |
| Q25 | Most others whose opinions I value would…, when I set firm boundaries in case of an incident of sexual violence. | Totally disapprove - Disapprove - Somewhat disapprove - Neither approve, nor disapprove - Somewhat approve  Approve - Totally approve |
| Q24 | How many people like you would set firm boundaries? | Nobody – Some people – About half of the people – Most people – Everybody |
| Q25 | I am … that if I want to, I can set firm boundaries in case of an incident of sexual violence | Not confident at all - Slightly confident  Moderately confident - Confident  Extremely confident |
| Q26 | Whether I set firm boundaries in case of an incident of sexual violence, is… | Not up to me - Slightly up to me - Moderately up to me - Mainly up to me  Completely up to me |
| Q27 | For me, setting firm boundaries in case of an incident of sexual violence is… | Very doubtful - Doubtful - Somewhat doubtful - Neither determined, nor doubtful - Somewhat determined – Determined - Very determined |
| Q28 | For me, setting firm boundaries in case of an incident of sexual violence is… | Very uncomfortable - Uncomfortable  Somewhat uncomfortable- Neither comfortable, nor uncomfortable- Somewhat comfortable - Comfortable - Very comfortable |
| Q29 | When I set firm boundaries in case of an incident of sexual violence, the situation will … | Worsen very much – Worsen – Rather worsen, Neither improve, nor worsen, Rather improve – Improve – Very much improve |
| Q30 | I find it …. that the situation improves. | Totally undesirable – Undesirable – Rather undesirable – Neither desirable, nor undesirable – Rather desirable, Desirable – Very desirable |
| Q31 | Most *fellow coaches* would…, when I set firm boundaries in case of an incident of sexual violence. | Totally disapprove - Disapprove - Somewhat disapprove - Neither approve, nor disapprove - Somewhat approve  Approve - Totally approve |
| Q32 | The *club board* would …, when I set firm boundaries in case of an incident of sexual violence. | Totally disapprove - Disapprove - Somewhat disapprove - Neither approve, nor disapprove - Somewhat approve  Approve - Totally approve |
| Q33 | I like to act like fellow coaches wants me to, regarding setting firm boundaries in case of an incident of sexual violence. | Not at all – Somewhat – Moderately - Very much - Extremely much |
| Q34 | I like to act like the club board wants me to, regarding setting firm boundaries in case of an incident of sexual violence. | Not at all – Somewhat – Moderately - Very much - Extremely much |
| Q35 | How many *fellow coaches* would set firm boundaries in case of an incident of sexual violence.? | None of the coaches – Some coaches – About half of the coaches – Most coaches – All coaches |
| Q36 | I want to be like my *fellow coaches* when it comes to setting firm boundaries in case of an incident of sexual violence. | Certainly not – Rather not – Maybe – Rather – Certainly |
| Q37 | The club having a code of conduct is … to set firm boundaries in case of an incident of sexual violence. | Totally not important - Slightly important - Moderately important - Important - Very Important |
| Q38 | My club has a code of conduct. | Certainly not – Probably not – Maybe – Probably – Certainly |
| Q39 | Being able to explain what is not allowed is … to set firm boundaries in case of an incident of sexual violence. | Totally not important - Slightly important - Moderately important - Important - Very Important |
| Q40 | I am able to explain what is (not) allowed in case of an incident of sexual violence. | Certainly not – Probably not – Maybe – Probably – Certainly |
| ***Target behavior 3: The coach intervenes in case of an incident of sexual violence*** | | |
| Q41 | I intend to intervene in case of a situation of sexual violence. | Absolutely not - Probably not - Maybe - Probably - Absolutely |
| Q42 | For me, intervening in case of an incident of sexual violence is … | Totally not important - Slightly important - Moderately important - Important - Very Important |
| Q43 | Most others whose opinions I value would…, when I intervene in case of an incident of sexual violence. | Totally disapprove - Disapprove - Somewhat disapprove - Neither approve, nor disapprove - Somewhat approve  Approve - Totally approve |
| Q44 | How many people like you would intervene in case of an incident of sexual violence? | Nobody – Some people – About half of the people – Most people – Everybody |
| Q45 | I am … that if I want to, I can intervene in case of an incident of sexual violence? | Not confident at all - Slightly confident  Moderately confident - Confident  Extremely confident |
| Q46 | Whether I intervene in case of an incident of sexual violence, is … | Not up to me - Slightly up to me - Moderately up to me - Mainly up to me  Completely up to me |
| Q47 | For me, intervening in case of an incident of sexual violence, is … | Very uncomfortable - Uncomfortable  Somewhat uncomfortable- Neither comfortable, nor uncomfortable- Somewhat comfortable - Comfortable - Very comfortable |
| Q48 | For me, intervening in case of an incident of sexual violence, is … | Very doubtful - Doubtful - Somewhat doubtful - Neither determined, nor doubtful - Somewhat determined – Determined - Very determined |
| Q49 | For me, feeling comfortable is | Totally not important - Slightly important - Moderately important - Important - Very Important |
| Q50 | For me, feeling determined is | Totally not important - Slightly important - Moderately important - Important - Very Important |
| Q51 | When I intervene in case of an incident of sexual violence, the situation will … | Worsen very much – Worsen – Rather worsen, Neither improve, nor worsen, Rather improve – Improve – Very much improve |
| Q52 | I find it …. that the situation improves. | Totally not important - Slightly important - Moderately important - Important - Very Important |
| Q53 | Most *fellow coaches* would…, when I intervene in case of an incident of sexual violence. | Totally disapprove - Disapprove - Somewhat disapprove - Neither approve, nor disapprove - Somewhat approve  Approve - Totally approve |
| Q54 | The *club board* would …, when I intervene in case of an incident of sexual violence. | Totally disapprove - Disapprove - Somewhat disapprove - Neither approve, nor disapprove - Somewhat approve  Approve - Totally approve |
| Q55 | I like to act like *fellow coaches* wants me to, regarding intervening in case of an incident of sexual violence. | Not at all – Somewhat – Moderately - Very much - Extremely much |
| Q56 | I like to act like the *club board* wants me to, regarding setting firm boundaries in case of an incident of sexual violence. | Not at all – Somewhat – Moderately - Very much - Extremely much |
| Q57 | How many *fellow coaches* would intervene in case of an incident of sexual violence? | None of the coaches – Some coaches – About half of the coaches – Most coaches – All coaches |
| Q58 | I want to be as my *fellow coaches* when it comes to intervening in case of an incident of sexual violence | Certainly not – Rather not – Maybe – Rather – Certainly |
| Q59 | The club having a code of conduct is…, when I need to intervene against the behavior of a fellow coaches. | Totally not important - Slightly important - Moderately important - Important - Very Important |
| Q60 | My club has a code of conduct. | Certainly not – Probably not – Maybe – Probably – Certainly |
| Q61 | Being able to explain what is not allowed is … when I need to intervene against a fellow coaches. | Totally not important – Slightly important – Moderately important – Important – Very Important |
| Q61 | I feel able to explain what is (not) allowed when I need to explain this to a fellow coaches. | Certainly not – Probably not – Maybe – Probably – Certainly |
| ***Target behavior 4: The coach reports the incident to the safeguarding officer*** | | |
| Q62 | I intend to report the incident to the safeguarding officer. | Absolutely not - Probably not - Maybe - Probably - Absolutely |
| Q63 | For me, report the incident to the safeguarding officer is … | Totally not important - Slightly important - Moderately important - Important - Very Important |
| Q64 | For me, report the incident to the safeguarding officer is … | Very uncomfortable - Uncomfortable  Somewhat uncomfortable- Neither comfortable, nor uncomfortable- Somewhat comfortable - Comfortable - Very comfortable |
| Q65 | Most others whose opinions I value would…, when I report the incident to the safeguarding officer. | Totally disapprove - Disapprove - Somewhat disapprove - Neither approve, nor disapprove - Somewhat approve  Approve - Totally approve |
| Q66 | How many people like you would report the incident to the safeguarding officers? | Nobody – Some people – About half of the people – Most people – Everybody |
| Q67 | I am … that if I want to, I can report the incident to the safeguarding officers? | Not confident at all - Slightly confident  Moderately confident - Confident  Extremely confident |
| Q68 | Whether I report the incident to the safeguarding officer is … | Not up to me - Slightly up to me - Moderately up to me - Mainly up to me  Completely up to me |
| Q69 | I feel … with regard to my fellow coaches, when I report the incident to the safeguarding officer. ` | Totally not loyal – not loyal – Somewhat loyal, nor not loyal – Somewhat loyal - Loyal – Very loyal |
| Q70 | For me, feeling loyal is … | Totally not important - Slightly important - Moderately important - Important - Very Important |
| Q71 | When I report the incident to the safeguarding officer, the situation will … | Worsen very much – Worsen – Rather worsen, Neither improve, nor worsen, Rather improve – Improve – Very much improve |
| Q72 | I find it …. that the situation improves. | Totally not important - Slightly important - Moderately important - Important - Very Important |
| Q73 | Most *fellow coaches* would…, when I report the incident to the safeguarding officer. | Totally disapprove - Disapprove - Somewhat disapprove - Neither approve, nor disapprove - Somewhat approve  Approve - Totally approve |
| Q74 | The *club board* would …, when I report the incident to the safeguarding officer. | Totally disapprove - Disapprove - Somewhat disapprove - Neither approve, nor disapprove - Somewhat approve  Approve - Totally approve |
| Q75 | I like to act like *fellow coaches* wants me to, regarding reporting the incident to the safeguarding officer. | Not at all – Somewhat – Moderately - Very much - Extremely much |
| Q76 | I like to act like *the club board* wants me to, regarding reporting the incident to the safeguarding officer. | Not at all – Somewhat – Moderately - Very much - Extremely much |
| Q77 | How many fellow coaches would report the incident to the safeguarding officer? | None of the coaches – Some coaches – About half of the trainer – Most trainer – All coaches |
| Q78 | I want to be as my fellow coaches when it comes to report the incident to the safeguarding officer. | Certainly not – Rather not – Maybe – Rather – Certainly |
| Q79 | The club having a safeguarding officer is … when it comes to report the incident. | Totally not important - Slightly important - Moderately important - Important - Very Important |
| Q80 | My club has a safeguarding officer. | Certainly not – Probably not – Maybe – Probably – Certainly |
| Q81 | It is important to be discrete, when I report the incident to the safeguarding officer. | Totally not important - Slightly important - Moderately important - Important - Very Important |
| Q82 | I feel able to be discrete, when I report the incident to the safeguarding officer. | Certainly not – Probably not – Maybe – Probably – Certainly |
| Q83 | If you want, you can leave here your suggestions or comments. | *Open text field* |
